# Prevalence and Awareness of Mucogingival Conditions and Deformities and Their Impact on Oral Health-Related Quality of Life Among Orthodontic Patients at a Tertiary Dental Care Center in Tamil Nadu, South India: A Questionnaire-Based Study

**DOI:** 10.7759/cureus.100027

**Published:** 2025-12-24

**Authors:** Chitra Girija Vallabhan, Arun Sadasivan, Steffi Vijayakumar, Nilima T. S., Jeiyasurya K, Athifa Jailani

**Affiliations:** 1 Department of Periodontology, Sree Mookambika Institute of Dental Sciences, Kulasekharam, IND

**Keywords:** awareness, frenum/abnormalities, gingiva/abnormalities, gingival recession, health-related quality of life, malocclusion, mucogingival deformities, prevalence, quality of life

## Abstract

Background

Mucogingival conditions and deformities (MGCDs) are frequently encountered around malposed teeth and may predispose individuals to attachment loss during or after orthodontic treatment. Therefore, a comprehensive pre-orthodontic evaluation of mucogingival status is essential to prevent potential complications. Enhancing patient awareness can reduce anxiety related to mucogingival corrective procedures and improve treatment acceptance. Furthermore, malocclusion can substantially influence oral health-related quality of life (OHRQoL). The aim of the present study was to assess the prevalence of specific MGCD by Angle's class of malocclusion, awareness of preexisting MGCDs, as well as OHRQoL, among orthodontic patients with different types of malocclusion.

Methodology

A cross-sectional questionnaire-based study was conducted among 150 pre-orthodontic patients (92 females and 58 males) within the age group of 15-35 years attending a private dental institution in Tamil Nadu, from October 2024 to December 2024. Patients were classified into Class I, Class II, and Class III malocclusion by Angle's classification. A pilot-tested and validated questionnaire comprising 14 closed-ended questions (Cronbach’s alpha = 0.86) was used to assess patients’ awareness of MGCDs. A clinical examination was performed to determine the presence of mucogingival deformities. OHRQoL was evaluated using the Oral Health Impact Profile-14 (OHIP-14) questionnaire. Statistical analyses were conducted using IBM SPSS Statistics for Windows, Version 27 (Released 2019; IBM Corp., Armonk, New York, United States). A p-value < 0.05 was considered statistically significant, and values < 0.01 were considered highly significant.

Results

The study revealed that the prevalence of mucogingival conditions was highest among individuals with Angle’s Class I malocclusion, followed by those with Class II and Class III malocclusion. However, no statistically significant association was observed between Class I, Class II, and Class III malocclusion and the presence of any mucogingival deformity (p > 0.05). The overall awareness of mucogingival conditions was low, with a mean awareness score of 5.2 ± 1.62 (median: 4.4) out of 14. While awareness of orthodontic tooth alignment was high (92.7%), recognition of mucogingival signs such as bleeding gums (27.3%), swollen gums (18.7%), and gingival thinning (16.7%) remained poor. The mean OHIP-14 score and its subscale scores varied across the different malocclusion groups, with the highest mean score recorded in Class II malocclusion (9.77 ± 8.83). Nevertheless, these differences were not statistically significant (p > 0.05).

Conclusion

Awareness of mucogingival deformities was generally poor, underscoring the need for targeted educational programs. Although the prevalence of MGCDs was more in Class I malocclusion compared to other malocclusions, the difference was not statistically significant. This underscores the need for a preorthodontic evaluation of mucogingival status in all patients seeking orthodontic treatment. The type of malocclusion did not appear to substantially influence OHRQoL, suggesting integration of psychological and emotional well-being of patients into comprehensive treatment planning.

## Introduction

Malocclusion is the third most prevalent oral health problem worldwide and is defined as any deviation from normal occlusion [1]. A 63.1% prevalence of malocclusion was reported in a study conducted in a certain part (Kanyakumari district) of Tamil Nadu, India, within its examined population [2].

Malposed teeth are often associated with alterations in the mucogingival complex and may predispose individuals to attachment loss during or after orthodontic treatment [3,4]. Mucogingival conditions and deformities (MGCDs) refer to defects in the morphology or position of the soft tissue and supporting bone around teeth. These result from deficiencies or structural irregularities in the periodontal tissues and include gingival recession (GR), inadequate keratinized tissue, thin gingival thickness (GT), shallow vestibular depth (VD), and aberrant frenum or muscle attachments [5]. MGCD poses periodontal, esthetic, and orthodontic challenges.

From a periodontal perspective, such conditions can lead to dental plaque accumulation, gingival inflammation, hypersensitivity, and root caries. Studies have shown that factors like inadequate keratinized tissue, a shallow vestibule, or a high frenal attachment promote food impaction, hinder oral hygiene maintenance, and increase the risk of GR in patients with occlusal anomalies [3,5]. Esthetically, an imbalance in the ratio of pink and white tissues may negatively affect self-esteem, confidence, and social interactions. Orthodontically, tooth movement in the buccal direction can predispose to the development or worsening of mucogingival deformities [6]. Hence, a thorough pre-orthodontic evaluation of mucogingival status is essential.

Improving awareness of preexisting mucogingival deformities can reduce patient anxiety and enhance acceptance of potential mucogingival corrective procedures. However, studies assessing awareness of MGCD among patients with different types of malocclusion are currently lacking.

The presence of malocclusion can negatively affect an individual’s self-perception throughout life and may lead to adverse social reactions as well as emotional and psychological distress [7]. Recently, oral health-related quality of life (OHRQoL) has been recognized by the World Health Organization as a key component of the Global Oral Health Programme and has gained increasing importance in both research and clinical practice [8]. OHRQoL is commonly assessed using the Oral Health Impact Profile (OHIP) and its shortened version, the OHIP-14, which comprises 14 questions across seven domains: functional limitation, physical pain, psychological discomfort, physical disability, psychological disability, social disability, and handicap [9].

Previous studies evaluating OHRQoL among individuals with different types of malocclusion have yielded inconsistent findings [7,10]. There is a paucity of available literature considering patient experiences and orthodontic treatment. Therefore, the present study aimed to assess the prevalence of specific MGCD by Angle's class of malocclusion, awareness of preexisting MGCDs, and OHRQoL across various malocclusion types.

## Materials and methods

This cross-sectional study was conducted among orthodontic patients in the age group of 15-35 years attending a tertiary care dental institution in Kanyakumari District, Tamil Nadu, from October 2024 to December 2024. Ethical approval was obtained from the Institutional Ethics Committee (SMIDS/IHEC No: 05/Protocol No: 11/2024). Written informed consent was obtained from all participants, with parental consent secured for participants younger than 18 years, and the confidentiality of personal data was assured.

Inclusion criteria comprised patients seeking orthodontic treatment in the age group of 15-35 years who could read and write in English and provided informed consent. This specific age group was chosen as it includes both adolescent and adult patients with distinct biological and psychosocial characteristics and varied treatment expectations. Hence, assessing their level of awareness of MGCD and OHRQoL contributes to the development of age-appropriate awareness programs and improves treatment outcomes. Exclusion criteria included patients undergoing orthodontic therapy, those requiring orthopedic or functional appliances, individuals with skeletal discrepancies requiring surgical corrections, individuals with mental illness/any systemic diseases, and those who refused to participate and submitted incomplete responses.

Sample size determination

The sample size for this study was calculated using the single-proportion formula: $n = \frac{Z^2 \times p \times (1 - p)}{d^2}$, where Z = 1.96 for a 95% confidence level, p = the anticipated proportion, and d = the absolute precision. For the awareness component, the anticipated proportion of awareness (p = 0.41) was obtained from a previously published study by Marusamy et al. in which the level of periodontal health awareness among orthodontic patients was 41% [11]. Using this value with a precision of d = 0.08, the estimated sample size was calculated as $n = \frac{(1.96)^2 \times 0.41 \times (1 - 0.41)}{(0.08)^2} = 145$. To account for a potential 5% non-response rate, the target sample size was increased to 152 participants. For the OHRQoL component, prior estimates of the mean and standard deviation of OHIP-14 scores within malocclusion subgroups were scarce in the literature for the target population; a conservative approach was adopted by using the larger calculated sample size from the awareness component to ensure adequate statistical power for intergroup comparisons of OHIP-14 scores using analysis of variance (ANOVA). Two participants did not complete the questionnaire and were excluded, resulting in a final analyzed sample of 150 participants.

Questionnaire development and data collection

A newly developed self-administered questionnaire was used in our study following an in-depth review of the literature and consultation with field experts (a committee of eight professors, comprising four from the Department of Periodontology and four from the Department of Orthodontics). It was pilot tested among 20 patients (excluded from the final analysis). Based on expert feedback, modifications were made, substituting technical MGCD terminology with layman-friendly terms to enhance clarity. These steps ensured the content validity of the questionnaire. Internal consistency for the awareness questionnaire was excellent, with a Cronbach’s alpha value of 0.859. The questionnaire was distributed manually to the participants, who were given 10 minutes to fill out the form (see Appendices).

The final questionnaire comprised three sections. The first section collected demographic details of the participants. The second section included 14 closed-ended binary-type (yes/no) questions with “Yes” responses (coded as 1) and “No" responses (coded as 0), which were summed to obtain the patients’ total awareness score. The third section evaluated OHRQoL using the short form of the OHIP-14. The OHIP-14 assesses OHRQoL across seven domains: functional limitation, physical pain, psychological discomfort, physical disability, psychological disability, social disability, and handicap. Responses were rated on a five-point Likert scale: 0 = never; 1 = hardly ever; 2 = occasionally; 3 = fairly often; 4 = very often/every day. Higher OHIP-14 scores indicated poorer OHRQoL [9].

**Figure 1:**
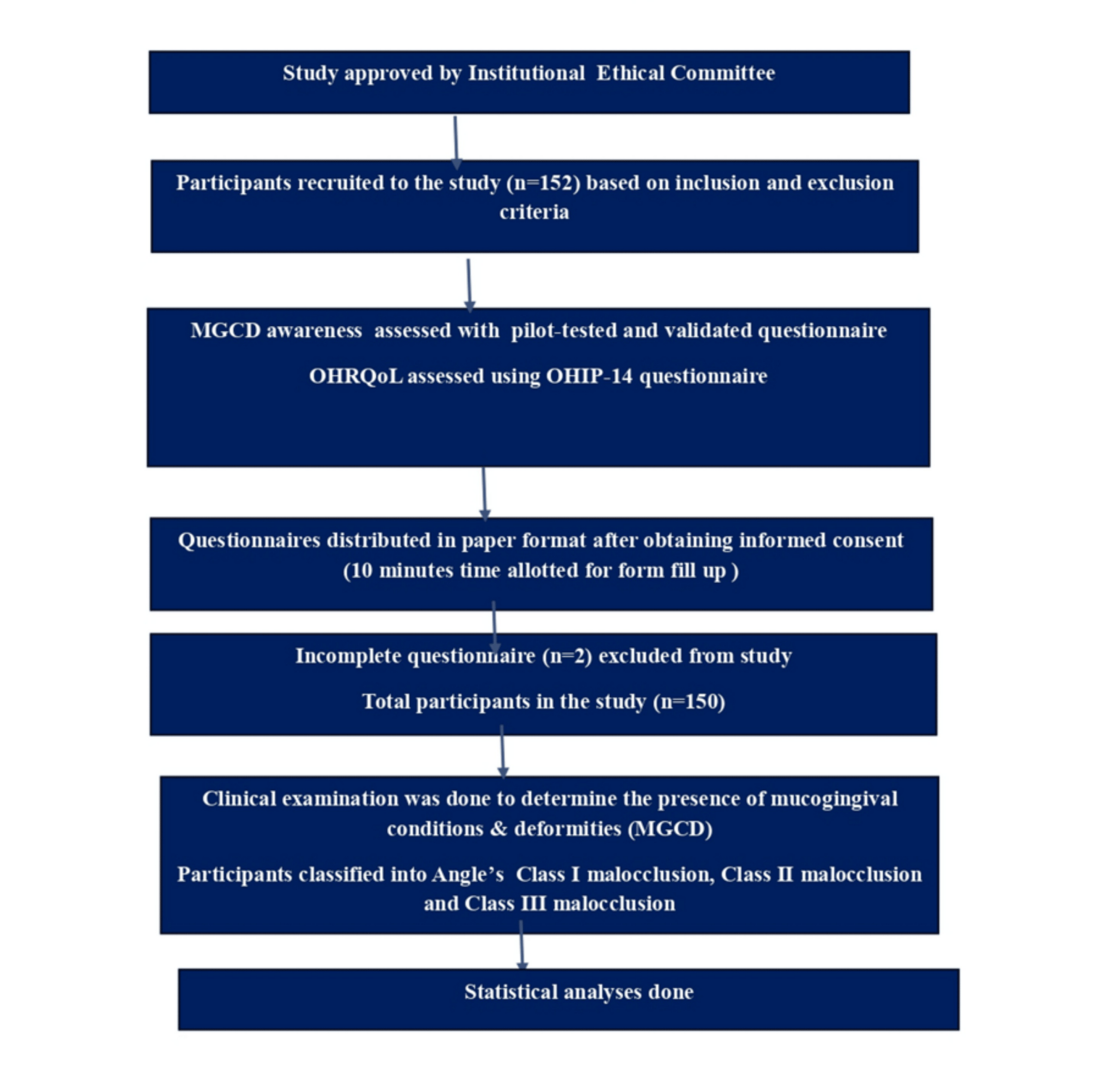
Flow diagram of the study. MGCDs: mucogingival conditions and deformities; OHRQoL: oral health-related quality of life; OHIP-14: Oral Health Impact Profile-14

An oral examination was conducted by a single calibrated examiner (JS) using a UNC 15 probe (Hu-Friedy, Chicago, IL, USA) to determine the presence of MGCDs. The examiner was calibrated under the supervision of an experienced periodontist (gold-standard examiner) by examining 10 volunteer patients with varying mucogingival conditions. Agreement on clinical parameters was assessed, discrepancies were resolved through discussion, and repeat examinations ensured consistency (Figure 1).

**Figure 2:**
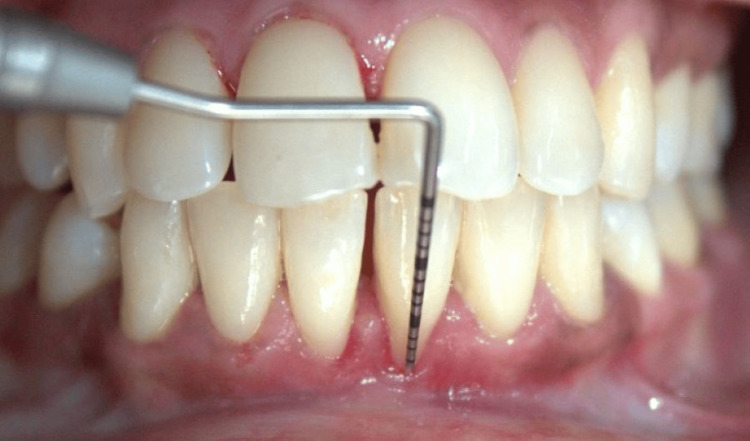
Gingival recession (GR) measured from the gingival margin to the cementoenamel junction (CEJ). Credit: Image obtained from one of the study participants.

GR, defined as the apical displacement of the gingival margin (GM) from the cemento-enamel junction (CEJ), was recorded as present or absent, considering only the maxillary and mandibular incisors (Figure 2).

**Figure 3:**
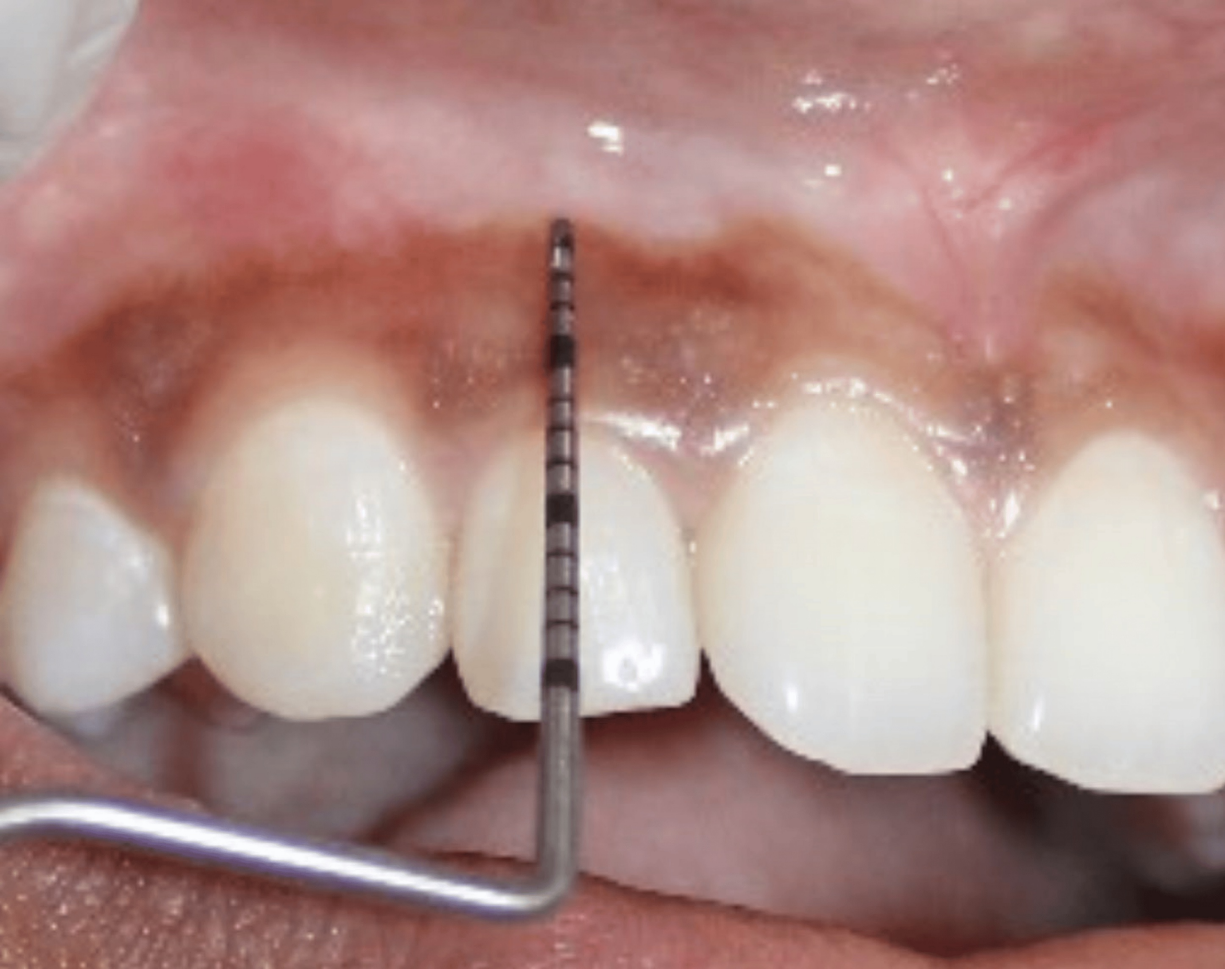
Width of keratinised gingiva (WKG) measured from the gingival margin to the mucogingival junction. Credit: Image obtained from one of the study participants.

The width of keratinized gingiva (WKG) was measured as the distance from the GM to the mucogingival junction and categorized as adequate (≥2 mm) or inadequate (<2 mm) (Figure 3) [5].

**Figure 4:**
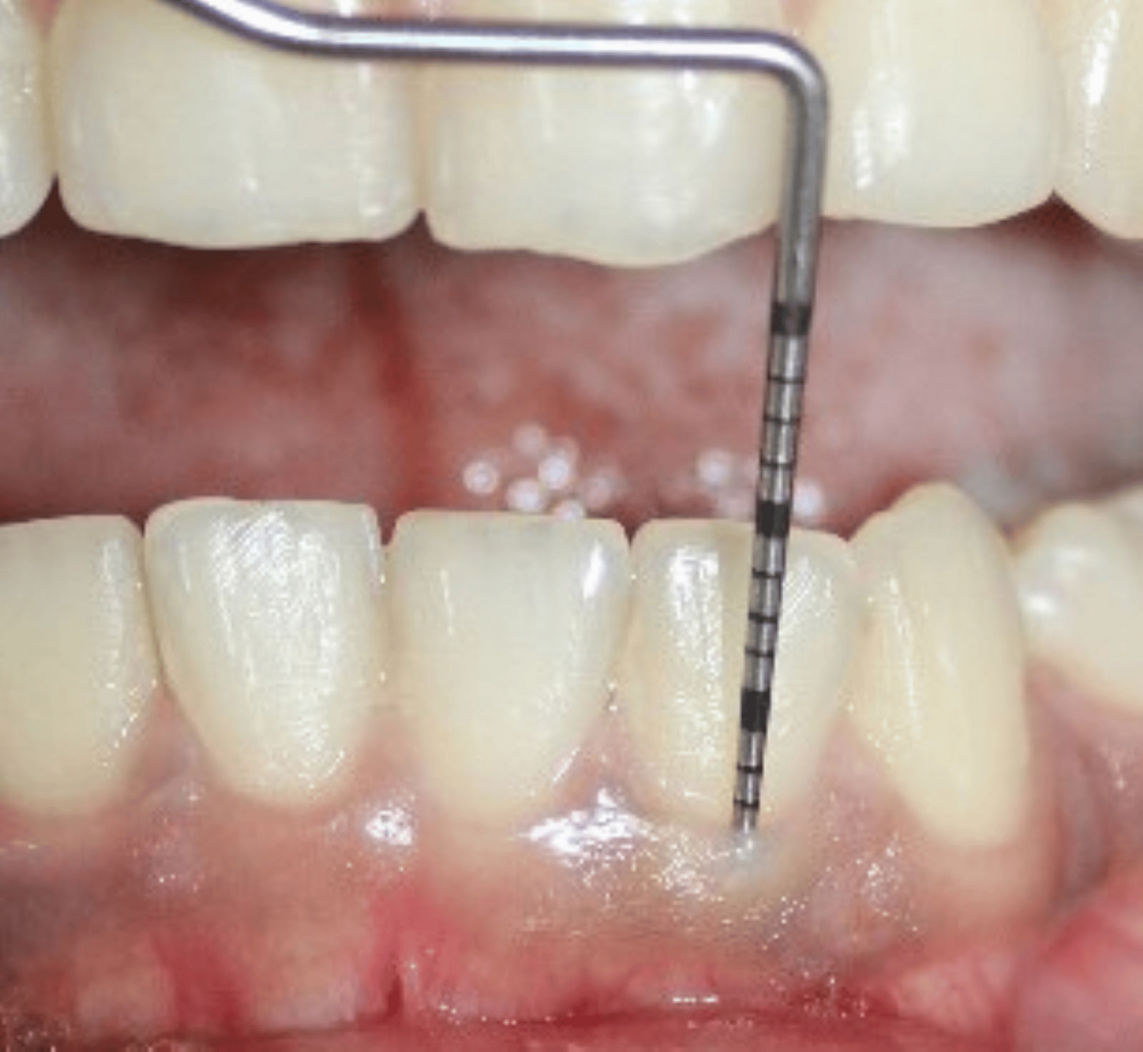
Probe visibility test used to determine gingival thickness. Credit: Image obtained from one of the study participants.

GT was assessed using the probe visibility test, with visibility of the probe through the gingiva indicating a thin biotype (Figure 4).

**Figure 5:**
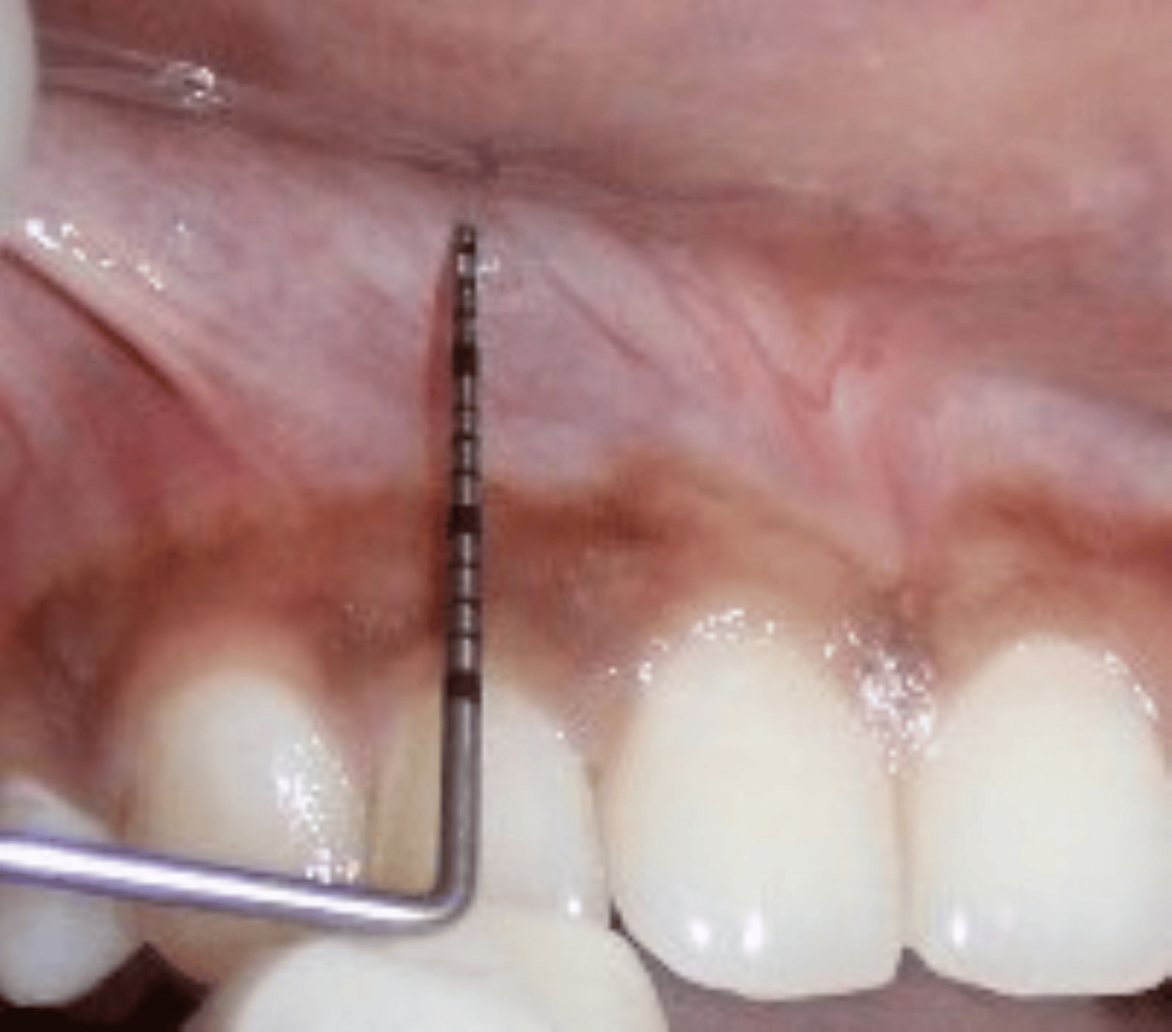
Vestibular depth (VD) measured as the distance from the free gingival margin to the deepest point of the mucolabial fold. Credit: Image obtained from one of the study participants.

Frenal attachment was classified as mucosal, gingival, papillary, or papilla-penetrating according to Placek’s classification; blanching or movement of the papillary tip during examination was considered indicative of an aberrant frenum. VD was measured as the distance from the free GM to the deepest point of the mucolabial fold and was recorded as adequate (>4 mm) or inadequate (≤4 mm) (Figure 5) [5].

Participants were categorized into Angle’s Class I, Class II, and Class III malocclusion groups based on the first molar relationship.

Statistical analysis

The statistical analysis was performed using IBM SPSS Statistics for Windows, Version 27 (Released 2019; IBM Corp., Armonk, New York, United States). The study utilized descriptive statistics for continuous variables (mean ± SD) and frequencies/percentages for categorical variables and employed the Kolmogorov-Smirnov test for normality assessment of continuous variables. The awareness score, primary outcome (range: 0-14), was computed by assigning a value of 1 to each “Yes” response and 0 to each “No” response across the 14-item awareness questionnaire. Since the summed score demonstrated an approximately normal distribution and met the assumptions of linear regression, it was analyzed as a continuous outcome variable. The secondary outcome of the study was the OHIP-14 score. Age group, gender, education level, and malocclusion type were treated as both exposure variables/predictors and potential confounders due to their independent influence on awareness level. All predictor variables were entered simultaneously using the enter method, enabling adjustment for potential confounding. A chi-square test was used to assess the association between mucogingival conditions and malocclusion types; multivariate linear regression was performed to identify predictors of total awareness score. The effect size for categorical variables was computed using Cramer's V. Intergroup comparisons of OHIP-14 scores across different malocclusion types were evaluated using one-way ANOVA. Missing data were checked prior to analysis. As missing responses were minimal (<0.4%), a complete-case analysis was performed without imputation. A p-value <0.05 was considered statistically significant, while p <0.01 was considered highly significant.

## Results

A total of 150 pre-orthodontic patients were enrolled in the study, comprising 92 females (61.3%) and 58 males (38.7%). The majority of participants were aged 16-25 years (n = 115, 76.7%), followed by 26-35 years (n = 27, 18.0%) and ≤15 years (n = 8, 5.3%). Most respondents had completed tertiary education (diploma, undergraduate, or postgraduate degree), accounting for 127 participants (84.7%). Among the study population, 76% exhibited Class I malocclusion, 17.3% Class II, and 6.7% Class III according to Angle’s classification. All participants resided in rural areas. The baseline characteristics of the participants are summarized in Table 1.

**Table 1:** Baseline demographic and clinical characteristics of the study participants (n = 150).

Parameters	Frequency (N)	Percentage distribution (%)
Age group		
≤15 years	8	5.3
16-25 years	115	76.7
26-35 years	27	18.0
Gender		
Male	58	38.7
Female	92	61.3
Education level		
No formal education	0	0
Primary and/or secondary school education	23	15.3
Tertiary education (diploma, undergraduate degree, postgraduate degree)	127	84.7
Type of malocclusion		
Angle’s Class I malocclusion	114	76.0
Angle’s Class II malocclusion	26	17.3
Angle’s Class III malocclusion	10	6.7

MGCDs were numerically most prevalent among individuals with Angle’s Class I malocclusion. Among patients presenting with GR, 62.5% were Class I, followed by 25.0% in Class II and 12.5% in Class III; however, this pattern showed no statistically significant association with malocclusion type (p = 0.277, Cramer’s V = 0.14, 95% confidence interval (CI): 0.00-0.27). Similarly, a thin gingival biotype was most frequently observed in Class I (72.2%) compared with Class II (21.1%) and Class III (6.7%), but showed no statistically significant association with malocclusion type (p = 0.268, V = 0.14, 95% CI: 0.00-0.27).

An inadequate WKG was also predominantly recorded in Class I (78.0%), followed by Class II (19.5%) and Class III (2.4%), but the association remained non-significant (p = 0.625, V = 0.10, 95% CI: 0.00-0.22). With respect to VD, the majority of inadequate cases (85.7%) occurred in Class I, with the remainder in Class II (14.3%) and none in Class III, reflecting no significant intergroup differences (p = 0.951, V = 0.04, 95% CI: 0.00-0.16).

For maxillary frenal attachment, the mucosal type was most common in Class I (82.0%), while papillary and papillary-penetrating variants occurred at lower frequencies across all groups; nevertheless, this distribution did not achieve statistical significance (p = 0.261, V = 0.14, 95% CI: 0.00-0.27). In the mandible, the mucosal frenal attachment was again most frequent in Class I (73.0%), followed by Class II (17.6%) and Class III (8.1%), with the difference remaining non-significant (p = 0.501, V = 0.11, 95% CI: 0.00-0.23).

**Figure 6:**
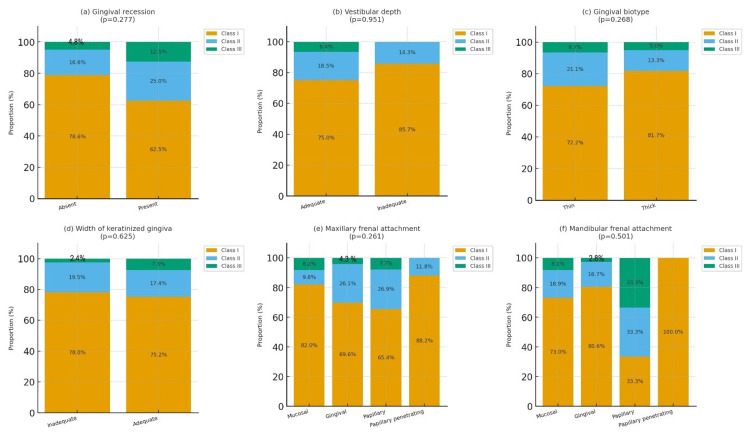
Bar graphs showing (a) gingival recession, (b) vestibular depth, (c) gingival biotype, (d) width of attached gingiva, (e) width of keratinized gingiva, (f) maxillary frenal attachment, and (g) mandibular frenal attachment across different malocclusion types.

Overall, although Angle’s Class I malocclusion demonstrated the highest numerical prevalence of most MGCDs, all effect sizes were negligible to small (Cramer’s V = 0.04-0.14), and the associated 95% CIs crossed zero, indicating that malocclusion type had no clinically meaningful relationship with mucogingival status (Table 2 and Figure 6).

**Table 2:** Distribution of mucogingival conditions and deformities across different malocclusions. A p-value < 0.05 indicates statistical significance. A p-value < 0.01 indicates high statistical significance. Statistical analysis was performed using the chi-square test.

Mucogingival conditions and deformities	Angle’s Class I	Angle’s Class II	Angle’s Class III	P-value	Effect size (Cramer’s V)	95% CI
Gingival recession				0.277	0.14	0.00-0.27
Absent	99 (78.6%)	21 (16.6%)	6 (4.8%)			
Present	15 (62.5%)	6 (25.0%)	3 (12.5%)			
Vestibular depth				0.951	0.04	0.00-0.16
Adequate	105 (75.0%)	26 (18.5%)	9 (6.4%)			
Inadequate	13 (85.7%)	1 (14.3%)	0 (0.0%)			
Gingival biotype				0.268	0.14	0.00-0.27
Thin	65 (72.2%)	19 (21.1%)	6 (6.7%)			
Thick	49 (81.7%)	8 (13.3%)	3 (5.0%)			
Width of keratinized gingiva				0.625	0.10	0.00-0.22
Inadequate	32 (78.0%)	8 (19.5%)	1 (2.4%)			
Adequate	82 (75.2%)	19 (17.4%)	8 (7.3%)			
Maxillary frenal attachment				0.261	0.14	0.00-0.27
Mucosal	50 (82.0%)	6 (9.8%)	5 (8.2%)			
Gingival	32 (69.6%)	12 (26.1%)	2 (4.3%)			
Papillary	17 (65.4%)	7 (26.9%)	2 (7.7%)			
Papillary penetrating	15 (88.2%)	2 (11.8%)	0 (0.0%)			
Mandibular frenal attachment				0.501	0.11	0.00-0.23
Mucosal	54 (73.0%)	14 (18.9%)	6 (8.1%)			
Gingival	58 (80.6%)	12 (16.7%)	2 (2.8%)			
Papillary	1 (33.3%)	1 (33.3%)	1 (33.3%)			
Papillary penetrating	1 (100.0%)	0 (0.0%)	0 (0.0%)			

Regarding awareness, 92 participants (61.3%) reported satisfaction with the appearance of their gums and smiles. Awareness of orthodontic treatment's role in tooth alignment was high, with 139 participants (92.7%) acknowledging it. In contrast, only 49 participants (32.7%) were aware that deleterious oral habits such as thumb sucking or teeth grinding could influence gingival position, and even fewer (40 participants, 26.7%) recognized that gum level could affect toothbrush placement. Awareness concerning gum bleeding (27.3%), swollen gums (18.7%), and sensitivity related to GR (30.0%) was also limited.

**Figure 7:**
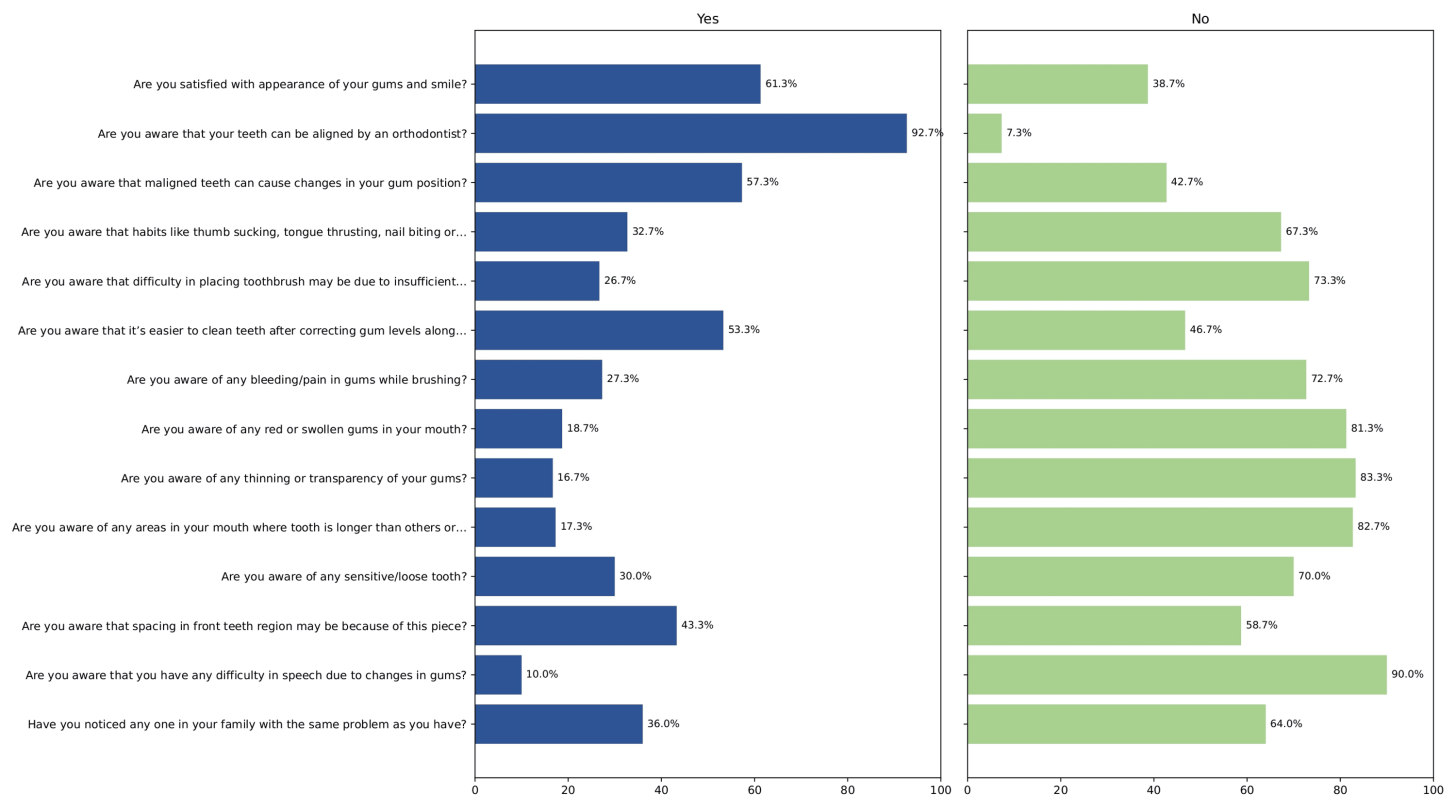
Awareness questionnaire items and corresponding response distribution among study participants (n = 150).

The overall level of awareness regarding mucogingival conditions was low among the study participants. The mean awareness score was approximately 5.2 ± 1.62 out of a possible 14, while the median score was 4.4, indicating that more than half of the participants recognized less than one-third of the awareness items. Based on predefined categories (0-4 = low, 5-9 = moderate, 10-14 = high), most participants fell within the low-to-moderate awareness range. Collectively, these findings indicate a considerable gap in mucogingival awareness among orthodontic patients in the present study (Figure 7).

According to the multivariate linear regression analysis, none of the demographic or clinical variables, including age group, gender, education level, or type of malocclusion, showed a statistically significant association with the awareness score (p > 0.05). However, participants with tertiary education demonstrated slightly higher awareness scores (β = 0.810 ± 0.541) (Table 3).

**Table 3:** Demographic and clinical predictors of awareness regarding mucogingival conditions and deformities (analysis performed using multivariate linear regression). Std. error: standard error of regression; t: t-test; p < 0.05 indicates statistical significance; p < 0.01 indicates high statistical significance

Variables/predictors	Beta coefficient (β)	Std. error	t statistic	P>|t|	95% confidence interval	
					Lower limit	Upper limit
Age group						
≤15 years	Baseline category					
16-25 years	-0.461	0.859	-0.54	0.592	-2.159	1.237
26-35 years	0.103	0.964	0.11	0.915	-1.802	2.008
Gender						
Male	Baseline category					
Female	-0.242	0.333	-0.73	0.468	-0.901	0.416
Education						
Primary and/or secondary school education	Baseline category					
Tertiary education (diploma, undergraduate degree, postgraduate degree)	0.810	0.541	1.50	0.137	-0.260	1.879
Malocclusion						
Angle’s Class I malocclusion	Baseline category					
Angle’s Class II malocclusion	-0.492	0.427	-1.15	0.250	-1.336	0.351
Angle’s Class III malocclusion	-0.414	0.676	-0.61	0.541	-1.750	0.921

Comparison of OHIP-14 scores across different malocclusion classes revealed that the overall mean score was highest among individuals with Angle’s Class II malocclusion (9.77 ± 8.83; 95% CI: 6.20-13.34), followed by Class I (9.40 ± 7.43; 95% CI: 8.02-10.78) and Class III (9.11 ± 6.13; 95% CI: 4.40-13.82). However, these differences were not statistically significant (p = 0.982), and the associated effect size was very small (η^2^ = 0.001), indicating that the type of malocclusion has minimal practical influence on overall OHRQoL as measured by the OHIP-14 score.

The mean psychological discomfort score was highest among Class III patients (1.61 ± 1.27; 95% CI: 0.63-2.59) and lowest among those with Class II malocclusion (1.33 ± 1.20; 95% CI: 0.85-1.81); however, this difference was not statistically significant (p = 0.885, η^2^ = 0.004). Functional limitation scores ranged from 0.17 ± 0.50 (95% CI: -0.21-0.55) in Class III to 0.56 ± 0.89 (95% CI: 0.20-0.92) in Class II, also demonstrating no significant variation (p = 0.488, η^2^ = 0.016). Similarly, physical pain scores were slightly higher in Class III (0.94 ± 0.81; 95% CI: 0.32-1.56) compared to Class I and II groups, but remained statistically non-significant (p = 0.698, η^2^ = 0.009).

**Figure 8:**
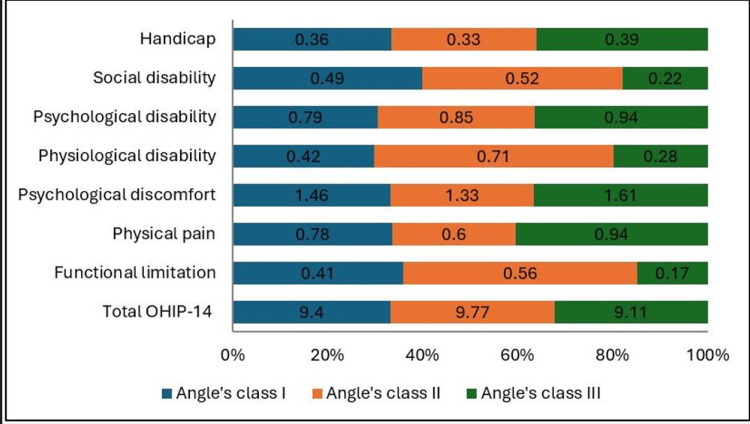
Graph showing total OHIP-14 scores and subscale scores across different malocclusion types. OHIP-14: Oral Health Impact Profile-14

Other OHIP-14 domains, including physical disability (p = 0.187, η^2^ = 0.031), psychological disability (p = 0.730, η^2^ = 0.0085), social disability (p = 0.623, η^2^ = 0.012), and handicap (p = 0.951, η^2^ = 0.002), also showed overlapping 95% CIs across groups, confirming an absence of meaningful intergroup differences. These findings indicate that although minor numerical variations exist across malocclusion categories, the overall OHRQoL remains broadly comparable with very small effect sizes, suggesting negligible clinical impact (Table 4 and Figure 8).

**Table 4:** Comparison of total OHIP-14 mean scores and OHIP-14 subscale scores by malocclusion classification. A p-value < 0.05 indicates statistical significance. A p-value < 0.01 indicates high statistical significance. Statistical analysis was performed using one-way analysis of variance (ANOVA). OHIP-14: Oral Health Impact Profile-14

Domain	Angle’s Class I, mean ± SD (95% CI)	Angle’s Class II, mean ± SD (95% CI)	Angle’s Class III, mean ± SD (95% CI)	Effect size η^2^	p-value
Total OHIP-14	9.40 ± 7.43 (8.02-10.78)	9.77 ± 8.83 (6.20-13.34)	9.11 ± 6.13 (4.40-13.82)	0.00117	0.982
Functional limitation	0.41 ± 0.62 (0.29-0.53)	0.56 ± 0.89 (0.20-0.92)	0.17 ±0.50 (-0.21 to 0.55)	0.01642	0.488
Physical pain	0.78 ± 0.92 (0.61-0.95)	0.60 ± 0.75 (0.30-0.90)	0.94 ±0.81 (0.32-1.56)	0.00914	0.698
Psychological discomfort	1.46 ± 1.20 (1.24-1.68)	1.33 ± 1.20 (0.85-1.81)	1.61 ±1.27 (0.63-2.59)	0.00432	0.885
Physical disability	0.42 ± 0.67 (0.30-0.54)	0.71 ± 0.89 (0.35-1.07)	0.28 ±0.36 (0.00-0.56)	0.03128	0.187
Psychological disability	0.79 ±0.85 (0.63-0.95)	0.85 ±0.86 (0.50-1.20)	0.94 ±0.58 (0.49-1.39)	0.00858	0.730
Social disability	0.49 ±0.67 (0.37-0.61)	0.52 ±0.89 (0.16-0.88)	0.22±0.36 (-0.06 to 0.50)	0.01227	0.623
Handicap	0.36 ±0.68 (0.23-0.49)	0.33 ±0.71 (0.04-0.62)	0.39 ±0.55 (-0.03 to 0.81)	0.00231	0.951

## Discussion

Existing literature provides limited evidence on the prevalence and awareness of preexisting MGCDs among orthodontic patients. The novelty of the present study lies in the assessment of preexisting MGCD across different types of Angle’s malocclusion. In addition, it provides insights into the OHRQoL of individuals with different malocclusion types.

**Figure 9:**
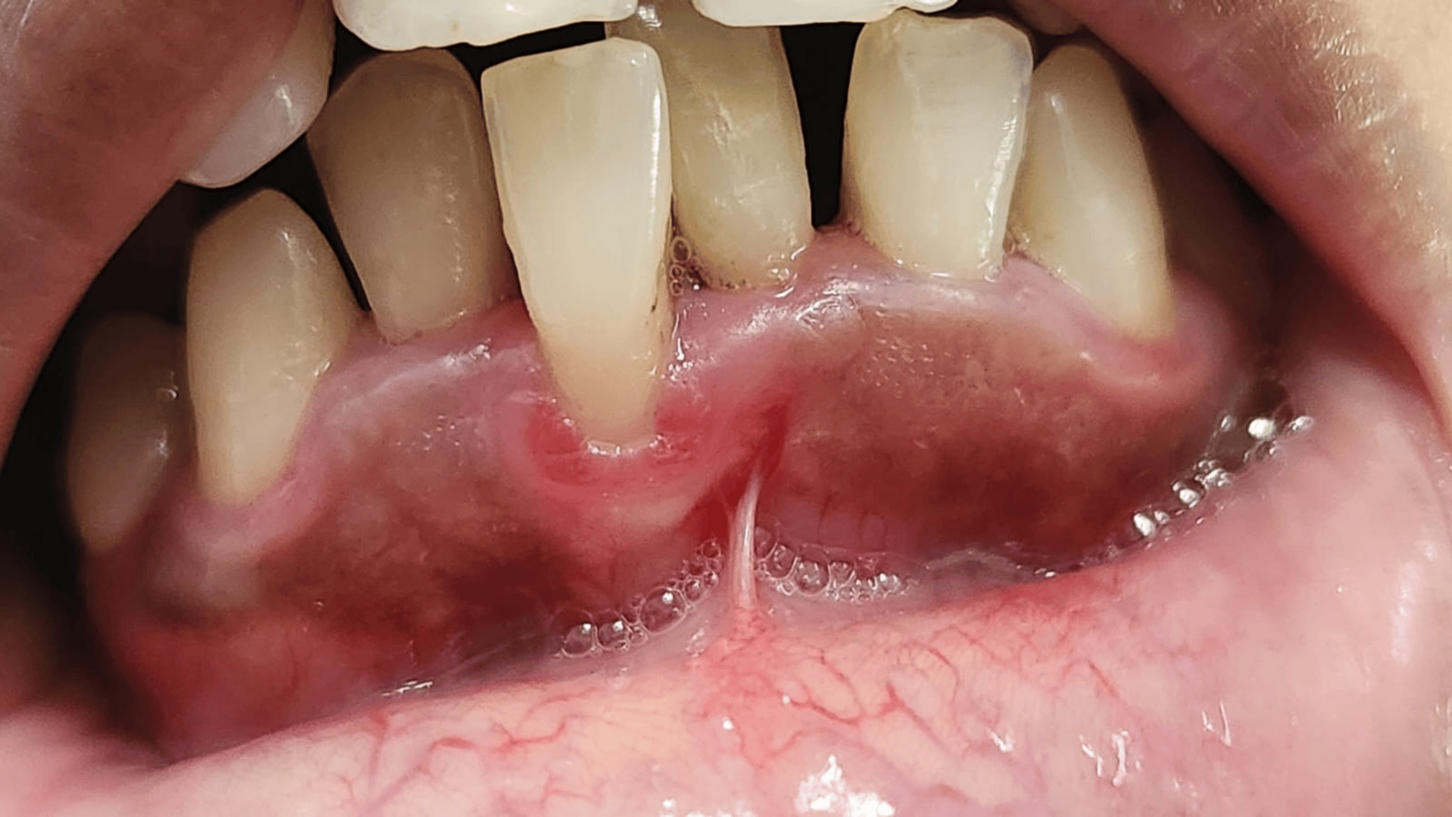
Gingival recession associated with a labially positioned incisor. Credit: Image obtained from one of the study participants.

Among the 150 participants, 76% presented with Angle’s Class I, 17.3% with Class II, and 6.7% with Class III malocclusion. Understanding the distribution of malocclusion within a population helps predict the likelihood of associated periodontal problems. The variation in malocclusion prevalence across studies can be attributed to genetic, environmental, and ethnic factors [12]. In our study, the prevalence of MGCD was highest in individuals with Class I malocclusion, followed by Class II and Class III. In the present study, GR was most prevalent among participants with Angle’s Class I malocclusion (62.5%). This finding contradicts reports by Menon et al., who described a high prevalence of GR in Class II Division I malocclusion, and by Ustun et al., who reported severe GR affecting the mandibular anterior teeth in a patient with Class III malocclusion [13,14]. This is likely due to differences in study design and population characteristics. However, no statistically significant association of GR with malocclusion type was found in our study (p = 0.277, Cramer’s V = 0.14, 95% CI: 0.00-0.27). The thin biotype was most prevalent among participants with Angle's Class I malocclusion (72.2%) and Class II (21.1%) subjects than Class III participants (6.7%). However, no significant association was found between GT and malocclusion types (p = 0.268, V = 0.14, 95% CI: 0.00-0.27), a finding supported by several population-based studies in adults [15-18] and a recent systematic review by Al-Thomali et al. [19] However, Sharma et al. [18] reported conflicting results, possibly due to variations in sample demographics and inclusion of skeletal malocclusions. Thin gingival phenotypes are more susceptible to recession during orthodontic tooth movement; hence, identifying and modifying the gingival phenotype (thin to thick) is crucial to prevent tooth movement-induced mucogingival complications (Figure 9) [5,19,20,21].

In our study, Angle’s Class I malocclusion also demonstrated the highest prevalence of inadequate WKG (78%), followed by Class II (19.5%) and Class III (2.4%), but the association remained non-significant (p = 0.625, V = 0.10, 95% CI: 0.00-0.22). This observation is consistent with previously published studies [16,17,21].

**Figure 10:**
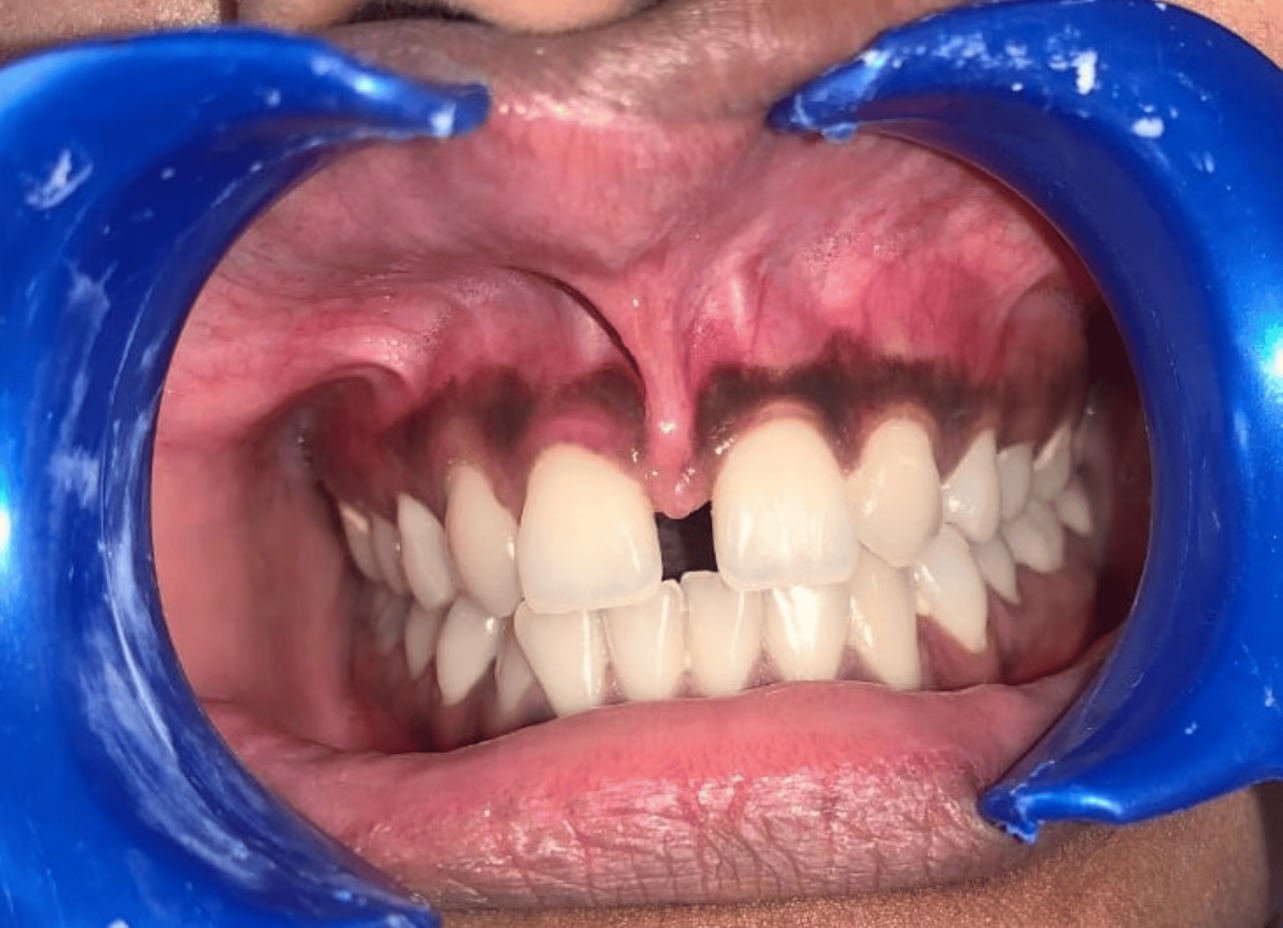
High maxillary labial frenum associated with a midline diastema. Credit: Image obtained from one of the study participants.

In the present study, the mucosal type of frenal attachment was most prevalent in the maxilla (82%), whereas the gingival type of attachment was predominant in the mandible (80.6%) among patients with Angle’s Class I malocclusion. These findings are consistent with the observations of Rajani et al. [22] but contradict those of Madi et al. [23], which may be attributed to differences in study population characteristics. High frenum attachments (papillary and papilla-penetrating types) were less prevalent among patients with Angle’s Class III malocclusion in our study, in contrast to the findings reported by Rajani et al. (Figure 10) [22]. The frenum distribution among the malocclusion types did not achieve statistical significance (p = 0.261, V = 0.14, 95% CI: 0.00-0.27).

Limited data exist regarding the prevalence of shallow vestibule in different types of malocclusion. Inadequate VD can compromise oral hygiene maintenance and contribute to plaque accumulation. In the present study, inadequate VD was the least prevalent of all MGCDs, occurring occasionally among Class I malocclusion patients (85.7%). This finding aligns with the results of Kus-Bartoszek et al. [24]. Notably, no cases of inadequate VD were observed among participants with Class III malocclusion, while 14.3% cases were noted in Class II malocclusion patients. But no significant intergroup differences were noted (p = 0.951, V = 0.04, 95% CI: 0.00-0.16).

**Figure 11:**
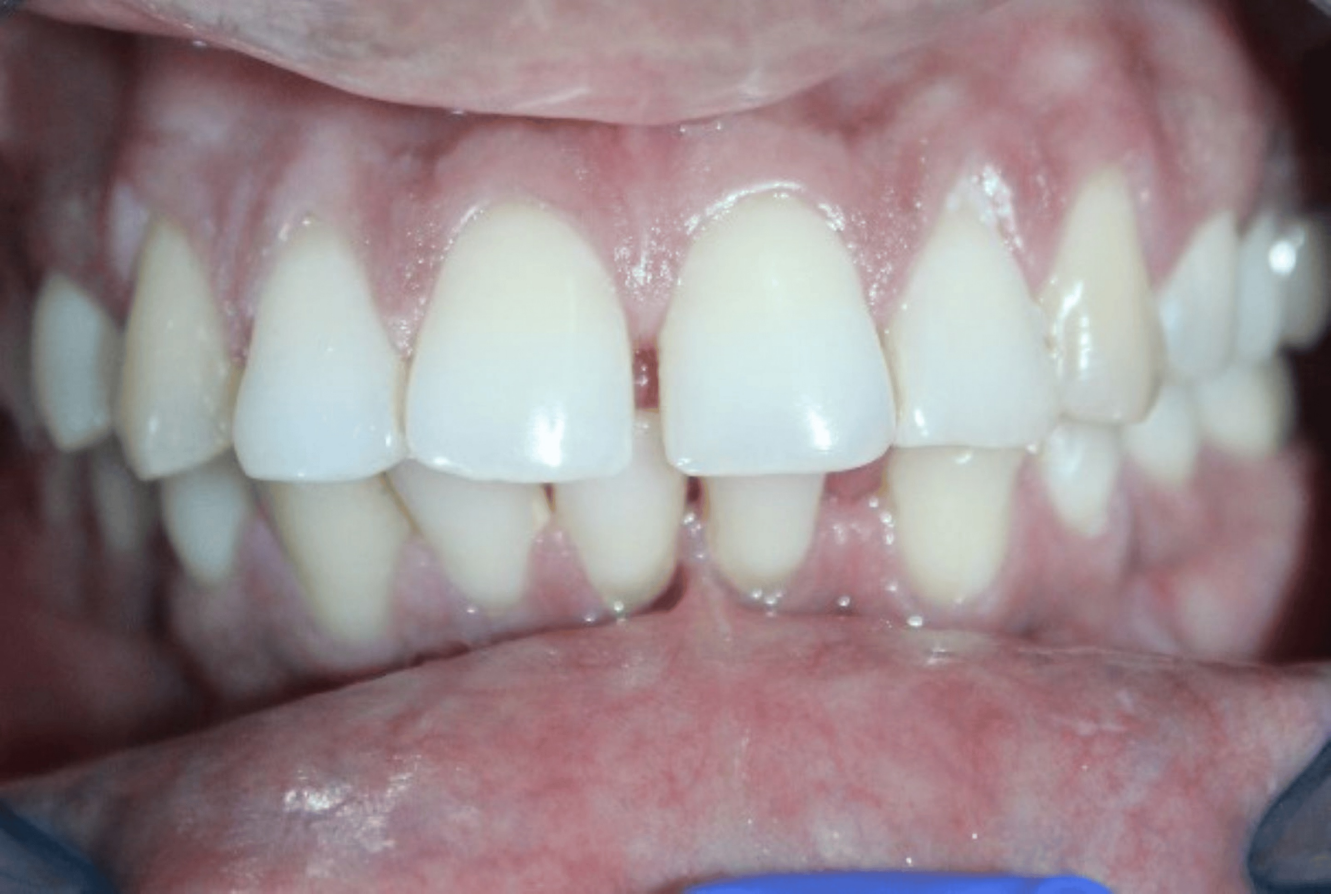
Inadequate vestibular depth associated with a high attached mandibular labial frenum. Credit: Image obtained from one of the study participants.

GR may be exacerbated by MGCDs, such as thin gingiva and inadequate keratinized tissue, during orthodontic treatment. Similarly, a shallow vestibule and high frenal attachment can interfere with effective oral hygiene practices, indirectly predisposing to periodontal disease (Figure 11). These findings underscore the importance of comprehensive mucogingival evaluation prior to initiating orthodontic therapy to minimize iatrogenic complications and enhance long-term periodontal health [5,6].

Thus, our study results showed no statistically significant association between MGCD and malocclusion types (p > 0.05). This finding is limited by small effect sizes, reduced statistical power, and a lack of assessment of the inclination of incisors.

In the present study, the majority of participants were aware that teeth alignment procedures are performed by orthodontists, a finding consistent with that of Sri et al. [25]. More than half of the participants recognized that correcting gum levels facilitates easier cleaning of teeth, in agreement with the observations of Yao et al. [26]. However, overall awareness regarding preexisting MGCDs was poor among orthodontic patients. To date, the literature on awareness of periodontal health, specifically in relation to MGCD, remains scarce. Hence, to the best of our knowledge, this study is the first to assess awareness of preexisting MGCD among pre-orthodontic patients.

In our study, graduates demonstrated slightly higher awareness scores regarding MGCD, supporting the well-established association between education level and health awareness outcomes [27]. Increased awareness of the potential adverse effects of MGCD on plaque control can help patients adopt better oral hygiene practices. Furthermore, improved awareness may reduce anxiety and enhance acceptance of possible mucogingival corrective procedures if required during orthodontic treatment.

Malocclusion and its management can both influence OHRQoL [7,10]. In the present study, participants with Angle’s Class II malocclusion exhibited comparatively poorer OHRQoL (9.77 ± 8.83; 95% CI: 6.20-13.34) than those with Class I (9.40 ± 7.43; 95% CI: 8.02-10.78) and Class III malocclusion (9.11 ± 6.13; 95% CI: 4.40-13.82), although this difference was not statistically significant (p = 0.982). This observation disagrees with Topal [28], who reported significantly higher OHRQoL values in Class III malocclusion patients (14.13 ± 6.20), probably due to differences in study populations and the inclusion of fewer severe Class III cases in the present study.

In the present study, Class III malocclusion was associated with higher subscale scores in the domains of psychological discomfort (1.61 ± 1.27; 95% CI: 0.63-2.59), physical pain (0.94 ± 0.81; 95% CI: 0.32-1.56), and psychological disability (0.94 ±0.58; 95% CI: 0.49-1.39). This may be attributed to the characteristic bite pattern of Class III patients, such as posterior crossbites, which can contribute to headaches, temporomandibular joint discomfort, and masticatory muscle pain. Additionally, the typical concave facial profile and anterior reverse bite may predispose individuals to psychosocial concerns, including anxiety regarding social appearance, embarrassment, and reduced self-esteem. Atik et al. reported higher levels of social appearance anxiety scores and lower levels of self-esteem scores in Class II and III malocclusions compared to Class I malocclusion [29].

Participants with Class II malocclusion exhibited comparatively higher subscale scores in functional limitation (0.56 ± 0.89; 95% CI: 0.20-0.92), physical disability (0.71 ± 0.89; 95% CI: 0.35-1.07), and social disability (0.52 ±0.89; 95% CI: 0.16-0.88) compared to Class I and Class III groups, suggesting greater difficulty with speech, mastication, and psychosocial adjustment. These issues are likely influenced by dental features such as proclined incisors, deep bite, spacing, and crowding. The characteristic convex profile associated with Class II malocclusion may also contribute to feelings of embarrassment and reduced social confidence. Palakolanu et al. [30] reported that patients with Class III malocclusion experienced the greatest difficulty in pronouncing sounds such as "s" and "t," followed by Class II (difficulty with "s" and "z") and Class I (difficulty with "s" and "sh").

In the current study, the scores in the psychological discomfort domain (p = 0.885, η^2^ = 0.004) and physical pain domain (p = 0.698, η^2^ = 0.009) were higher in Class III compared to Class I and II groups, but remained statistically non-significant (p > 0.05). For the remaining OHIP-14 domains - physical disability (p = 0.187, η^2^ = 0.031), psychological disability (p = 0.730, η^2^ = 0.009), social disability (p = 0.623, η^2^ = 0.012), and handicap (p = 0.951, η^2^ = 0.002) - overlapping 95% CIs indicated an absence of meaningful intergroup differences. This is in contrast to the results reported by Topal [28]. Thus, we found that OHIP-14 subscale scores varied across malocclusion types, but the overall impact on perceived OHRQoL did not significantly differ based on malocclusion type. However, this finding is limited by small effect sizes and reduced statistical power. Our study emphasizes the importance of OHRQoL evaluation alongside pre-orthodontic mucogingival status evaluation in malocclusion patients. This integrated approach helps to better understand patient expectations and achieve better patient-centric outcomes (psychosocial and emotional well-being).

Strengths and limitations

The strengths of our study were assessing mucogingival deformities, which are seen in orthodontic patients, a pilot-tested and validated instrument to assess MGCD awareness, a calibrated single examiner, and a well-established OHRQoL measure (OHIP 14). Nonetheless, inclusion of orthodontic patients (only English literates) attending a single rural tertiary care center may have introduced selection bias and limited the generalizability of the findings. To overcome this, multicentric studies with larger sample sizes are recommended. The absence of adjustment for oral hygiene or periodontal status, lack of assessment of skeletal relationships, growth pattern of study participants (age range 15-35 years) (potential confounders), and uneven distribution of participants within the malocclusion subgroup (reduces statistical power and increases type II error) are additional concerns. Only Angle’s molar relationship was used as the clinical criterion for classification, while dental factors such as deep bite, crossbite, and skeletal discrepancies were not considered. Furthermore, the correlation between MGCDs and OHRQoL was not assessed.

## Conclusions

The prevalence of MGCDs varied across malocclusion types and was highest among individuals with Angle’s Class I malocclusion, underscoring the importance of a thorough pre-orthodontic assessment of mucogingival status. No significant association was found between malocclusion type and the prevalence of MGCD. Awareness regarding MGCD was generally poor among study participants, underscoring the need for targeted educational programs. Furthermore, the type of malocclusion did not appear to substantially influence overall OHRQoL, although variations were observed across individual OHIP-14 domains. Evaluating OHRQoL enables orthodontic specialists to better integrate the psychological and emotional well-being of the patients into comprehensive treatment planning.

## Appendices

Questionnaire for assessing awareness of mucogingival conditions and deformities among orthodontic patients

1. Are you satisfied with appearance of your gums and smile? Yes/no

2. Are you aware that your teeth can be aligned by an orthodontist? Yes/no

3. Are you aware that maligned teeth can cause changes in your gum position? Yes/no

4. Are you aware that habits like thumb sucking, tongue thrusting, nail biting or teeth grinding can cause changes in your gum position? Yes/no

5. Are you aware that difficulty in placing toothbrush may be due to insufficient gum level along with irregularly placed teeth? Yes/no

6. Are you aware that its easier to clean teeth after correcting gum levels along with aligning teeth? Yes/no

7. Are you aware of any bleeding /pain in gums while brushing? Yes/no

8. Are you aware of any red or swollen gums in your mouth? Yes/no

9. Are you aware of any thinning or transparency of your gums? Yes/no

10. Are you aware of any areas in your mouth where tooth is longer than others or gums are pulling away from your teeth? Yes/no

11. Are you aware of any sensitive/loose tooth? Yes/no

12. Are you aware that spacing in front teeth region may be because of thin piece of tissue that connects lip to gums? Yes/no

13. Are you aware that you have any difficulty in speech due to changes in gum level along with irregular teeth? Yes/no

14. Have you noticed any one in your family with the same problem as you have? Yes/no
